# The role of gut vascular barrier in experimental alcoholic liver disease and A. muciniphila supplementation

**DOI:** 10.1080/19490976.2020.1851986

**Published:** 2020-12-01

**Authors:** Christoph Grander, Felix Grabherr, Ilaria Spadoni, Barbara Enrich, Georg Oberhuber, Maria Rescigno, Herbert Tilg

**Affiliations:** a Department of Internal Medicine I, Gastroenterology, Hepatology and Endocrinology, Medical University Innsbruck, Innsbruck, Austria; b Department of Biomedical Sciences, Humanitas University, Milan, Italy; c INNPATH, Institute of Pathology, University Hospital of Innsbruck, Innsbruck, Austria; d Humanitas Clinical and Research Center-IRCCS, Via Manzoni 56, 20089 Rozzano, Milan, Italy

**Keywords:** Gut vascular barrier, alcoholic liver disease, Akkermansia muciniphila

## Abstract

The translocation of bacterial components from the intestinal lumen into the portal circulation is crucial in the pathogenesis of alcoholic liver disease (ALD). Recently the important role of the gut vascular barrier (GVB) was elucidated in alcoholic liver disease. Here we report about the influence of *A. muciniphila* supplementation in experimental ALD on the GVB. Ethanol feeding was associated with increased Pv-1, indicating altered endothelial barrier function, whereas *A. muciniphila* administration tended to restore GVB. To further investigate GVB in experimental ALD, β-catenin gain-of-function mice, which display an enhanced GVB, were ethanol-fed. β-catenin gain-of-function mice were not protected from ethanol-induced liver injury, suggest an alternative mechanism of ethanol-induced GVB disruption. The description of the GVB in ALD could pave the way for new therapeutic options in the future.

## Introduction

With great interest we read the article by Maccioni L. et al.,^1^ describing the role of intestinal permeability and microbial composition in human alcoholic liver disease (ALD). Particularly the description of gut-vascular barrier (GVB) alterations in ALD caught our attention. ALD displays the hepatic manifestation of alcohol overconsumption, associated with steatosis, hepatitis (alcoholic hepatitis, AH) and progression to fibrosis, cirrhosis and hepatocellular carcinoma.^2,3^ The pathogenesis of ALD is tightly linked to the gut-liver axis, encompassing ethanol-induced gut barrier disruption and translocation of bacterial components into the liver.^4^ As we could show previously, intestinal microbiota composition and especially the abundance of *A. muciniphila* is associated with gut barrier function in alcoholic liver disease. Chronic alcohol overconsumption resulted in diminished *A. muciniphila* levels. Administration of live *A. muciniphila* decreased endotoxinemia and enhanced gut barrier function.^5^ Beside the well-known epithelial barrier function, Spadoni I. et al.^6^ recently illuminated the gatekeeper functions of intestinal vascular endothelia. The GVB controls the translocation of antigens and prohibits the entry of bacteria into the blood stream. Here we report the assessment of GVB in experimental ALD.

## Results

**Figure 1.**
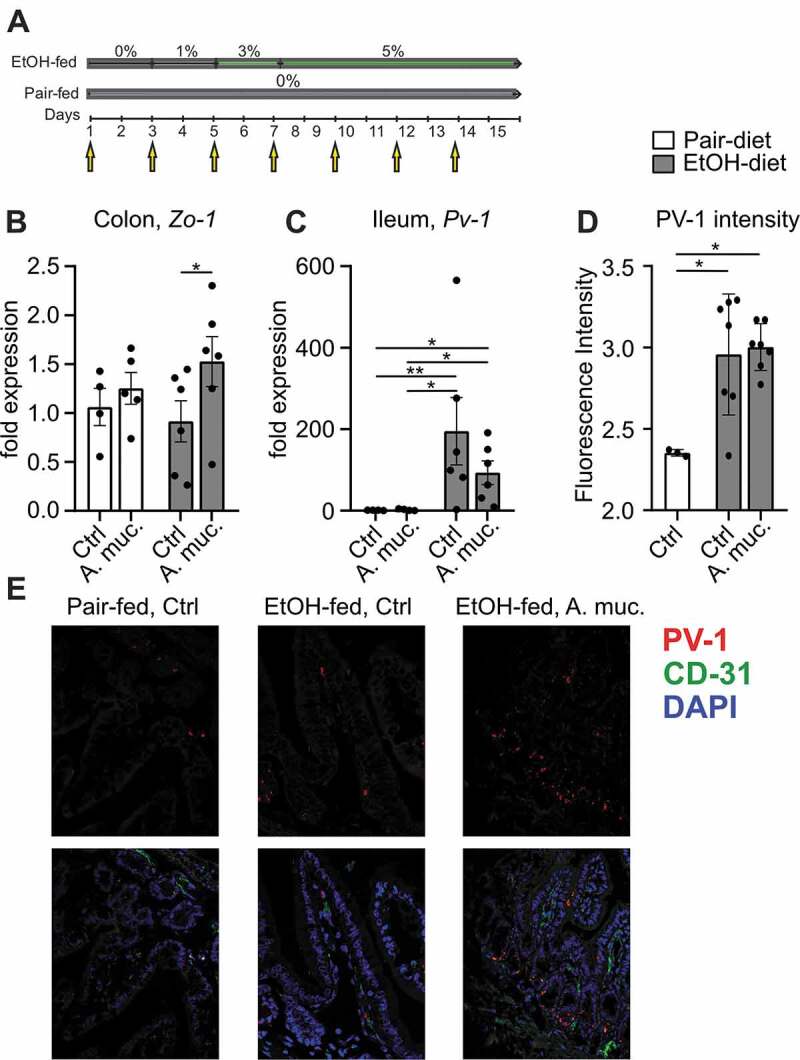
(a) Graphical illustration of experimental design. (b) Expression of Zo-1 in colon samples and (c) *Pv-1* expression in ileum samples of pair-fed and ethanol-fed mice supplemented with *A. muciniphila*. (d) Analysis of PV-1 fluorescence intensity of ileal slides and (e) representative pictures. *p < .05; **p < .01; ***p < .001 according to Kruskal-Wallis test with uncorrected Dunn’s test (b-c) and one-way ANOVA with Tukey’s post hoc test (d). A. muc, Akkermanisa muciniphila; Ctrl, control; EtOH, ethanol; Pv-1, plasmalemma vesicle associated protein, Zo-1, Zonula occludens-1

Seven to eight weeks old female WT mice were fed an ethanol-containing Lieber-DeCarli diet or control diet for 15 days. *A. muciniphila* was administered every other day (Figure 1a). Ethanol feeding decreased colonic expression of *Zo-1* compared to pair-fed mice, whereas *A. muciniphila* tended to restore *Zo-1* expression (Figure 1b). Ethanol-fed mice displayed significantly increased ileal expression levels of *Pv-1 (*plasmalemma vesicle associated protein) compared to pair-fed mice. Administration of *A. muciniphila* was associated with a 47% decreased *Pv-1* expression in ethanol-fed mice, although this effect was not significant (Figure 1c). Moreover, ethanol-fed mice showed an increased staining index of PV-1 compared to pair-fed controls (Figure 1d,e).

**Figure 2.**
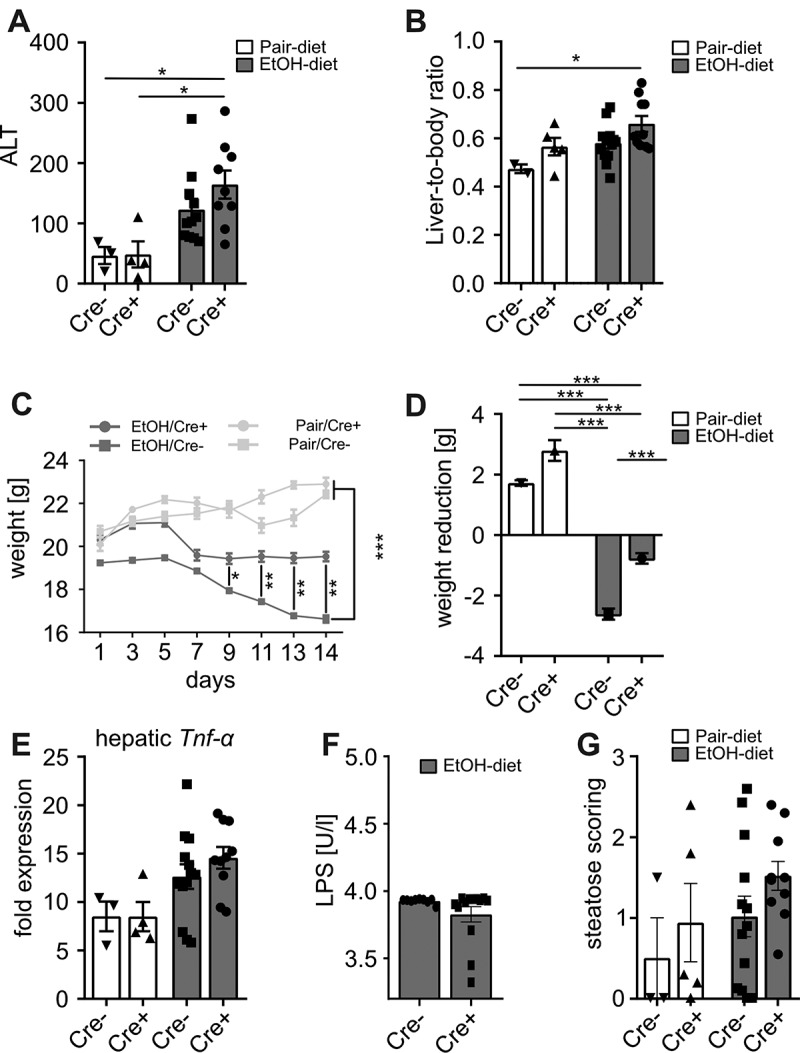
Shown are (a) ALT levels and (b) the liver-to-body ratio as markers for liver injury. (cd) Ethanol-fed Cre+ (gain of function) mice showed less weight loss compared to ethanol-fed controls. (e) Hepatic expression analysis of *Tnf-α*. (f) Analysis of serum lipopolysaccharide levels in ethanol-fed mice. (g) Scoring of hepatic steatosis by a professional pathologist based on h&e liver sections. *p < .05; **p < .01; ***p < .001 according to Kruskal-Wallis test followed by Dunn’s multiple comparison test (a, g) or uncorrected Dunn’s multiple comparison test (e), one-way ANOVA (b-d) and Mann-Whitney test (f). ALT, alanine aminotransferase; EtOH, ethanol; h&e, hamatoxylin and eosin; LPS, lipopolysaccharide; Tnf-α, tumor necrosis factor alpha

To further investigate GVB in experimental ALD, β-catenin gain-of-function mice, which display an enhanced GVB, were fed a Lieber-DeCarli diet for 15 days. Ethanol-fed gain-of-function mice (Cre+) showed no significant difference in alanine aminotransferase (ALT) levels (Figure 2a) and liver-to-body ratio (Figure 2b) compared to littermates (Cre-). Ethanol feeding was associated with weight-loss in Cre- mice, whereas Cre+ mice were protected from weight-loss. Pair-fed Cre+ mice tended to gain more weight compared to pair-fed Cre- mice (Figure 2c,d). Hepatic expression of *Tnf-α* was higher in ethanol-fed controls compared to pair-fed animals, whereas no significant difference could be observed between Cre+ and Cre- mice (Figure 2e). Likewise, endotoxin levels were comparable between ethanol-fed Cre+ and Cre- mice (Figure 2f). Moreover, hepatic liver sections were analyzed by a professional pathologist (G.O.). Ethanol-fed mice tended to show increased hepatic steatosis, whereas we could not observe any difference between Cre+ and Cre- mice (Figure 2g).

## Discussion

The translocation of bacterial components is an essential factor in the pathogenesis of ALD.^4,7,8^ The importance of barrier structures like the mucus layer, anti-microbial products and epithelial barrier to prevent the translocation of bacterial products have been described in the past.^9,10^ Recently the central role of the GVB and endothelia as a gate keeper for bacterial products was described in experimental nonalcoholic steatohepatitis (NASH). In models of experimental NASH and cirrhosis PV-1 activation was increased.^11,12^ Important to note, also patients with NASH showed an altered GVB compared to controls.^12^

In our work we could underpin the findings of Maccioni L et al.,^1^ describing the leakiness of gut vascular endothelia in experimental ALD. Likewise, we used Pv-1 (an integral membrane protein associated to the diaphragms of endothelial fenestrae) as a marker of endothelial barrier dysfunction. Ileal expression of *Pv-1* was increased in ethanol-fed mice. Interestingly, *A. muciniphila* administration seemed to be associated with lower *Pv-1* expression levels, which could indicate a restored GVB. As shown previously,^5^ *A. muciniphila* administration resulted in restored epithelial gut barrier function, displayed by increased expression of *Zo-1*, an important tight junction protein. The relationship between epithelial barrier and GVB seems unclear and needs to be addressed in future studies.

In a second step we assessed liver injury in a genetic model with mice displaying an enhanced GVB. Surprisingly these mice were only protected from weight-loss, but showed similar features of liver injury compared to controls. Although others linked the disruption of GVB with the WNT/β-catenin signaling pathway,^6,12^ our data could suggest an alternative mechanism of ethanol-induced GVB disruption. It was shown recently that endotoxin stimulation^13^ as well as systemic inflammation induce Pv-1 activation.^14,15^ These findings suggest that ethanol-induced systemic inflammation could result in endothelial barrier dysfunction and *A. muciniphila* might beneficially influence the GVB by reducing endotoxinemia and hepatic inflammation.

## Material and methods

### Mouse experiments

All experiments were aligned to ethical principles according to legal law (BMWFW-66.011/0019-WF/V/3b/2015). To study the relationship between experimental ALD and *A. muciniphila* supplementation, seven to eight weeks old female WT mice were fed a Lieber-DeCarli diet^16,17^ containing 1–5 vol% (EtOH-fed) ad libitum for 15 days. *A. muciniphila* (1,5x109 CFU/200 µl PBS) or vehicle (PBS) was by intragastric infusion with a 24-gauge stainless steel free tube every other day, starting on day 1. All animals were anesthetized before exsanguination and tissue sampling.

Beta-catenin gain-of-function mice were kindly provided by Prof. Maria Rescigno. Crossed β-catenin^lox(ex3)/lox(ex3)^ and Cdh5(PAC)-CreERT2 mice were used^6,18,19^ to obtain a mouse line in which the exon 3 of β-catenin gene (Catnnb) was deleted in endothelial cells in an inducible manner. To induce the Cre recombination and β-catenin stabilization, Tamoxifen (4 mg per mouse, MP Biomedicals, Santa Ana, CA, USA) was orally gavaged on four days prior to ethanol feeding (as described above).

### Immunofluorescence staining

Slides were prepared as described before.^5^ Primary antibodies anti-murine Plvap (BD, Franklin Lakes, NJ, USA, BD 553849) and anti-mouse CD31 (Abcam, Cambridge, UK, ab 124432) were used. Next, slides were incubated with secondary goat antibodies: anti-rabbit antibody (AF488, life technologies, Carlsbad, CA, USA) and anti-rat antibody (AF 568, life technologies, Carlsbad, CA USA). Slides were mounted with Prolong® Diamond Antifade Mountant supplemented with DAPI (4′,6′-diamidino-2-phenylindole, life technologies, Carlsbad, CA, USA) and analyzed with a 340 confocal microscope (Zeiss, Oberkochen, Germany). Intensity was quantified by two independent, blinded observers in three randomly picked fields of view.

### Liver histology

H&e staining was performed by the Institute of Pathology at the Medical University of Innsbruck. A pathologist (G.O.) analyzed the h&e liver sections regarding hepatic steatosis.

### RNA isolation and measurement

RNA from liver tissue was purified by homogenization of samples in TRIzol® reagent (Thermo Fisher Scientific, Waltham, MA, USA). Reverse transcription was accomplished with the Reverse Transcription System (Thermo Fisher Scientific, Waltham, MA), followed by qPCR. For qPCR SybrGreen (Eurogentec, Seraing, Belgium) and the Mx3000 qPCR cycler (Stratagene California, CA) were used. PCR primers sequences are available upon request.

### Statistical analysis

For analyzing our data, we used GraphPad PRISM 5 (La Jolla, California, USA). Mann-Whitney test, Kruskal-Wallis test followed by uncorrected Dunn’s multiple comparison test or one-way analysis of variance followed by post hoc Tukey test were used where appropriate. Two or more independent experiments were performed for each modality. Results are shown as mean±SEM. Statistical significance was considered at *p* < .05.
